# mDia1 senses both force and torque during F-actin filament polymerization

**DOI:** 10.1038/s41467-017-01745-4

**Published:** 2017-11-21

**Authors:** Miao Yu, Xin Yuan, Chen Lu, Shimin Le, Ryo Kawamura, Artem K. Efremov, Zhihai Zhao, Michael M. Kozlov, Michael Sheetz, Alexander Bershadsky, Jie Yan

**Affiliations:** 1 Mechanobiology Institute, National University of Singapore, Singapore, 117411 Singapore; 2 Department of Physics, National University of Singapore, Singapore, 117542 Singapore; 3 Department of Physiology and Pharmacology, Sackler Faculty of Medicine, Tel Aviv University, Tel Aviv, 69978 Israel; 4 Department of Biological Sciences, Columbia University, New York, NY 10027 USA; 5 Department of Molecular Cell Biology, Weizmann Institute of Science, Rehovot, 76100 Israel; 6 Centre for Bioimaging Sciences, National University of Singapore, Singapore, 117546 Singapore

## Abstract

Formins, an important family of force-bearing actin-polymerizing factors, function as homodimers that bind with the barbed end of actin filaments through a ring-like structure assembled from dimerized FH2 domains. It has been hypothesized that force applied to formin may facilitate transition of the FH2 ring from an inhibitory closed conformation to a permissive open conformation, speeding up actin polymerization. We confirm this hypothesis for mDia1 dependent actin polymerization by stretching a single-actin filament in the absence of profilin using magnetic tweezers, and observe that increasing force from 0.5 to 10 pN can drastically speed up the actin polymerization rate. Further, we find that this force-promoted actin polymerization requires torsionally unconstrained actin filament, suggesting that mDia1 also senses torque. As actin filaments are subject to complex mechanical constraints in living cells, these results provide important insights into how formin senses these mechanical constraints and regulates actin organization accordingly.

## Abstract (web summary)

Formins are actin-polymerisation factors that are sensitive to force. Here the authors find that pulling force on an actin filament promoted faster actin polymerisation by the formin mDia1, and also found that the actin filament must be torsionally unconstrained, suggesting that mDia1 can also sense torque.

## Introduction

The actin cytoskeleton is involved in various physiological and pathological functions, such as cell migration, differentiation, embryo development, and cancer metastasis^1,2^. The highly dynamic organization of the actin cytoskeleton is tightly controlled by a variety of proteins that regulate actin nucleation, polymerization, de-polymerization, branching, bundling, and localization^3,4^. The formin family of proteins plays critical roles in promoting nucleation and regulating actin polymerization. Formin forms a homodimer through its homology FH2 domains, and these dimerized FH2 domains form a ring-like shape, which encircles an actin filament at the barbed end^5^. This FH2 ring slows down the recruitment of actin monomers at the barbed end compared with free barbed ends^5,6^. Therefore, the FH2 ring acts as a “gate”, imposing an energy barrier for adding new actin monomers to the filaments.

The N-terminus of each FH2 monomer is linked to an intrinsically disordered FH1 domain containing multiple polyproline tracks that interact with the actin-binding protein profilin with an affinity of 3.4–17.8 μM^7,8^. Profilin has a high affinity to actin monomers (*K*_D_ ~ 0.1 μM^9–13^), therefore, the two FH1 domains of a formin homodimer enrich the local concentration of actin monomers near the vicinity of the barbed end of actin filaments, facilitating actin polymerization. Collectively, actin polymerization depends on the complex interplay between the formin FH1 domain that promotes actin polymerization and the FH2 ring that suppresses actin polymerization.

It has been believed that the inhibitory “closed” ring of the FH2 domain has to be “opened” up to allow recruitment of new actin monomers^14,15^. In vivo, actin filaments are subject to mechanical stretch generated by actomyosin contraction^16^, and the resulting tensile force is transmitted to formin in the case of formin-dependent actin polymerization^15,17^. It raises an appealing possibility that force may perturb the FH2 conformation and facilitate the closed-to-open transition of the FH2 ring. If this is the case, formin would be a mechanosensing protein that regulates actin polymerization in a force-dependent manner. If the closed-to-open transition of the FH2 ring is accompanied with a positive extension change Δ, then under force it will be associated with a negative potential energy change of −*F*Δ (the scalar *F* denotes the magnitude of force), which biases the probability ratio of the open state over the closed state by an exponential factor of exp(−*βF*Δ) (*β* denotes (*k*_B_ *T*)^−1^. Based on this principle, Kozlov et al.^18,19^ predicted that force should strongly promote actin polymerization, which is hereafter referred to as the mechanical gating mechanism.

In contrast to this prediction, a recent single-molecule experiment showed that in the absence of profilin, the budding yeast formin Bni1p-dependent actin polymerization in 1.5 μM G-actin was inhibited by sub picoNewton (pN) forces: the G-actin on-rate decreased from ~13 μM^−1^ s^−1^ at zero force to <4 μM^−1^ s^−1^ at forces larger than 0.4 pN. While in the presence of 2.5 μM profilin and 1.5 μM G-actin, pN forces slightly accelerated polymerization^20^. Increasing force from 0 pN to near 1.5 pN, the G-actin on-rate only increased <50%. Jégou et al.^15^ showed that forces in the range of 1–3 pN applied to formin mDia1 in the presence of profilin strongly promoted actin polymerization, with a G-actin on-rate of ~83 μM^−1^ s^−1^ at a force of around 3 pN in the presence of 3 μM G-actin and 3 μM profilin. In the latter work, the authors did not perform experiments in the absence of profilin, therefore it remains unclear whether force promotes or inhibits actin polymerization for mDia1-mediated actin polymerization without profilin. The observation of the force inhibition of Bni1p-dependent actin polymerization by force in the absence of profilin conflicts with the predicted mechanical gating mechanism of the FH2 ring. It also raises a question whether this inhibition is specific to Bni1p or universal to other formin family proteins.

Previous structural studies have predicted that formin rotates relative to the F-actin filament around the filament axis^5^, and this has been demonstrated in the case of mDia1ΔN3-dependent polymerization^21^. In living cells actin filaments are highly cross-linked and therefore unable to freely rotate^22^, therefore it is the formins that undergo rotation in vivo during progressive polymerization of actin filaments. Previous studies have suggested that actin rotates clockwise relative to formin looking along the direction of elongating filament^21^ (alternatively, formin rotates relative to the filament in a right-hand manner looking along the reverse direction). If the relative rotation between formin at the barbed end and the superparamagnetic bead attached to actin filament is inhibited, torsion would accumulate in the filament during actin polymerization, which could possibly suppress actin polymerization. The occurrence of rapid, progressive actin polymerization in cells implies that cells employ some mechanisms to relieve the accumulated torsion during actin polymerization. The exact mechanism by which the torsion stress is relieved remains unclear.

In order to provide new insights into formin-mediated actin polymerization, we recorded the extension change of single-actin filaments under different forces using magnetic tweezers in the absence of profilin. We find that forces of a few pN applied to mDia1ΔN3 can drastically speed up the polymerization of actin filaments when they are torsionally unconstrained. Our theoretical analysis indicates that the force-induced acceleration of torsion-unconstrained actin polymerization can be explained by the reduction of energy cost needed for the closed-to-open transition of the FH2 ring, along with additional reduction of the critical concentration required of actin monomers for polymerization. Together, our results directly support the gating mechanism of the FH2 domain. In addition, the rotational slippage between the FH2 ring and the actin filament, should it happen in vivo, would require involvement of other accessory proteins. The biological implications of these findings and the possible causes of the contradiction between our results and those reported for the Bni1p-dependent actin polymerization^20^ are discussed.

## Results

### Applying constant forces to F-actin with magnetic tweezers

**Fig. 1:**
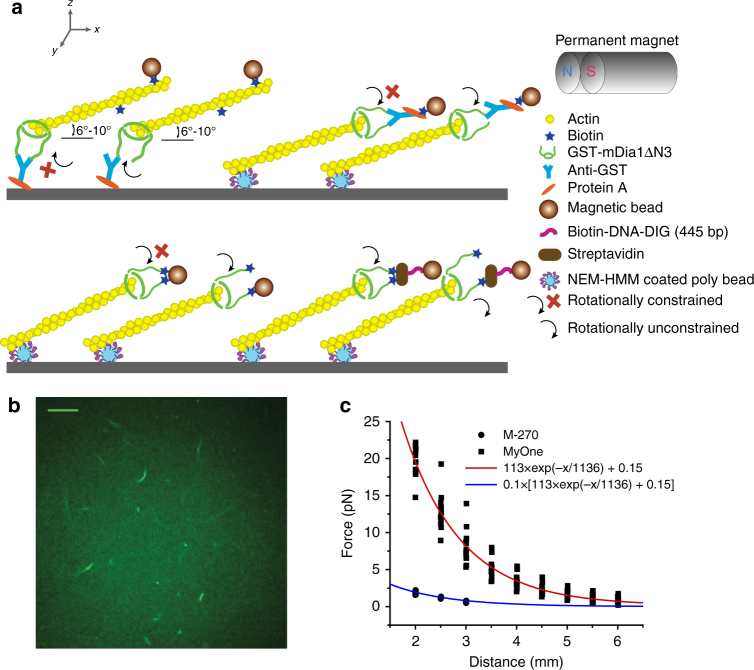
Schematics of experimental set-up and force calibration. **a** Actin filaments are tethered in four different designs using two differently tagged mDia1ΔN3. The GST-mDia1ΔN3-bound barbed end of the filament is either attached to an anti-GST–Protein A coated coverslip surface (top-left) or to an anti-GST–Protein A coated superparamagnetic bead (top right). The biotin-mDia1ΔN3-bound barbed end is attached to a streptavidin-coated superparamagnetic bead either directly (bottom left) or indirectly (bottom right) through a torsionally unconstrained DNA linker. The actin filament is tethered to an immobilized NEM-HMM-coated bead on a coverslip through a point on the actin filament somewhere near the tipped end. The actin filament was stretched at 6–10° above the surface using transverse magnetic tweezers. The rotationally unconstrained or constrained tethers are indicated. **b** Fluorescence imaging revealed sparsely distributed seeding filaments on the surface under our preparation conditions. Scale bar represent 10 μm. **c** The calibrated force-distance profiles for 2.8-μm-diameter M270 beads and 1.0-μm-diameter MyOne beads. 12 beads for M270 and 10 beads for MyOne

Tensile forces were applied to actin filaments tethered in several different ways using a transverse magnetic tweezers set-up (Fig. 1a). N-terminal GST-tagged and biotin-tagged mDia1ΔN3, indicated as GST-mDia1ΔN3 and biotin-mDia1ΔN3, respectively, were used in this study. In all cases, the mDia1ΔN3 were bound to pre-polymerized short actin filaments (with a typical length of a few μm) stabilized with phalloidin. These short actin filaments served as “seeds” for further polymerization upon introduction of G-actin solution (500 nM) (Fig. 1b).

GST-mDia1ΔN3 was attached to the corverslip surface or to the superparamagnetic bead surface through a GST–anti-GST–protein A-surface linkage. When attached to the coverslip, the seeding filaments were prepared at a concentration of 10% biotinylated G-actin, which allowed attachment of a streptavidin-coated superparamagnetic bead (1-μm-diameter MyOne bead or 2.8-μm-diameter M-270 Dynal beads, Invitrogen) somewhere near the tipped end. When GST-mDia1ΔN3 was attached to the anti-GST coated superparamagnetic bead, the actin filament was linked to an immobilized NEM-HMM coated polybead on the coverslip surface via a region somewhere near the tipped end of the actin filament. Biotin-mDia1ΔN3 was attached to a streptavidin-coated superparamagnetic bead either directly or indirectly through a torsionally unconstrained DNA linker. The actin filament was tethered to an immobilized NEM-HMM coated polybead on a coverslip. Force was applied at a few degrees above the surface to the filament and mDia1ΔN3 through the superparamagnetic bead using a pair of magnets. These different tethering methods and force exertion are illustrated in Fig. 1a.

Each mDia1ΔN3 dimer carries two tags at the N-termini of the two FH1 domains, therefore, either one of the FH1 domains or both are linked to an anti-GST–Protein A (in the case of GST-mDia1ΔN3) or to a streptavidin (in the case of biotin-mDia1ΔN3). An anti-GST provides two GST-binding sites, however, the chance that both FH1 domains in a GST-mDia1ΔN3 are bound might be limited due to potential steric interaction between the bulky GST tag (26 kDa). In contrast, a streptavidin contains four biotin-binding sites that can interact with the small biotin tag at the very end of the FH1 domain. Therefore, the chance that both FH1 domains in a biotin-mDia1ΔN3 are bound to a streptavidin should be much higher. The possibilities of single-FH1 or double-FH1 tethering are also illustrated in Fig. 1a.

Since the rotation of the superparamagnetic bead is inhibited by the magnetic tweezers, the actin filament will be rotationally constrained in the case of double-FH1 tethering, while it is rotationally unconstrained in the case of single-FH1 tethering. It was predicted^5^ and confirmed^21^ that during polymerization the FH2 domain will rotate around the axis of the actin filament. Therefore, torsion stress will build up when the relative rotation between FH2 and actin is inhibited (i.e., during double-FH1 tethering). Whether rotational constraints can suppress formin-mediated actin polymerization or whether there is a mechanism employed by formin to avoid accumulation of torsion stress have not yet been investigated. Using these differently tethered actin filaments, we investigated the effects of force dependence on mDia1ΔN3-mediated actin polymerization under rotationally unconstrained and constrained conditions. After a tether bound with a single-superparamagnetic bead was identified, force was applied and the movement of the superparamagnetic bead was recorded. In order to minimize possible bead-surface and filament-surface interactions, force was applied at a direction of 6–10° above the surface, so that the bead-surface separation increases as the filament elongates (Supplementary Note 2).

For a given superparamagnetic bead, the force **F** = *F* **z** applied to the bead is solely dependent on the bead-magnets separation *s*, where **z** denotes the direction of force. At the same value of *s*, due to heterogeneity in their magnetization, different superparamagnetic beads may have different force values, which differ from each other with a relative error of *α* ~ 20% among the 2.8-μm-diameter superparamagnetic beads, and *α* ~ 15% among the 1-μm-diameter superparamagnetic beads (Fig. 1c). Therefore, *F*(*s*) was carefully calibrated from a set of bead-magnets separations over a force range up to 20 pN. At each value of *s*, the forces applied to 10–15 beads were measured by recording the drifting speeds of the beads in 90% (volume fraction) glycerol solution (Methods section). The average of the force, $\bar F(s)$, can be fitted with a single-exponential decay function (Fig. 1c). In actin polymerization experiments, the force was determined based on the calibrating curve as $\bar F(s) \pm \alpha \bar F(s)$.

The change of the position of the bead along the force direction is indicative of actin elongation. Under forces of a few pN, a tether had a typical life time in the order of a couple of minutes. The breakage of a single tether resulted in sudden increase of the moving speed of the bead to the level of a free drifting bead at the same force (>10 μm s^−1^ at ~1 pN near the surface (Supplementary Fig. 2)). In rare cases where there are more than one tethered actins between the bead and the surface, each breakage caused a sudden change in the bead position, which could be easily distinguished from the breakage of a single tether. Using this method, we sought to quantify the force dependence of the GST-mDia1ΔN3-mediated polymerization of single-actin tethers with well-calibrated forces and minimized non-specific actin-surface or bead-surface interactions.

### GST-mDia1ΔN3-mediated actin polymerization under force

**Fig. 2:**
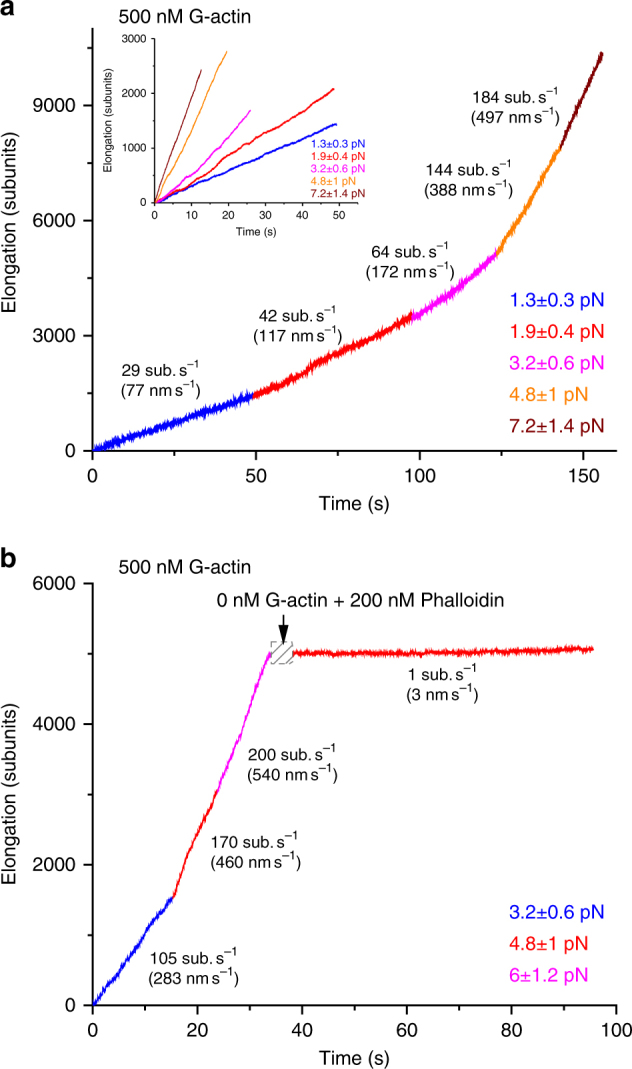
Actin elongation speed is force-dependent. **a** Elongation trajectories of a single-F-actin filament in 500 nM G-actin solution under different forces. **b** The elongation of the F-actin filament stopped after free G-actin was washed off. The number of replication of the force-dependent actin polymerization at each force is indicated in Fig. 4

**Fig. 3:**
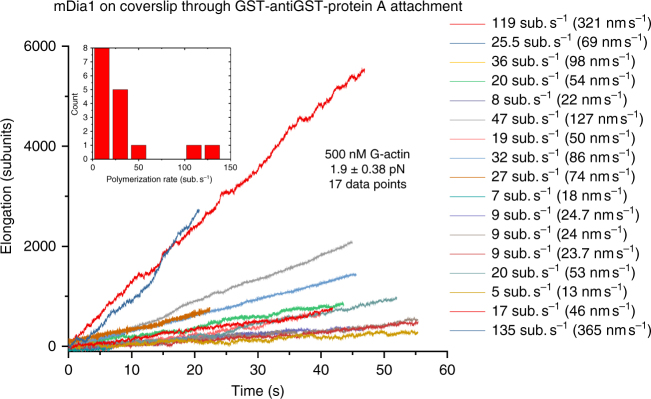
The F-actin elongation rate varies depending on tether. The elongation trajectories of F-actin in 500 nM G-actin solution at 1.9 ± 0.4 pN obtained from 17 different tethers show a wide variation in speed. The inset shows the histogram of the elongation rates based on the 17 tethers

**Fig. 4:**
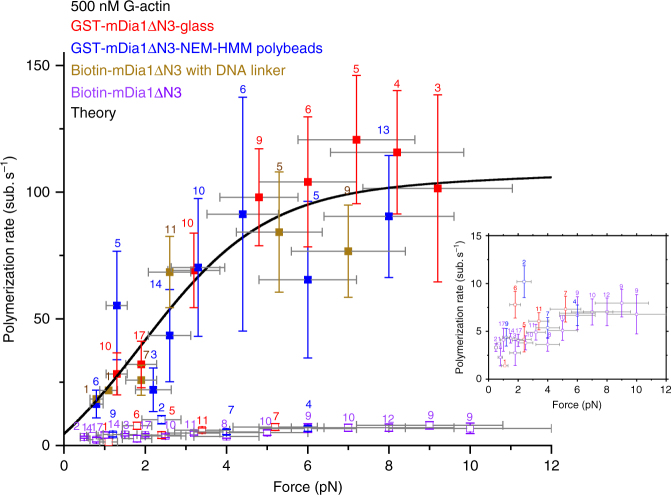
Force-dependent F-actin elongation rate varies depending on tether configuration. The data show the actin elongation speed as a function of force for cases where GST-mDia1ΔN3 was attached to coverslip surface (red solid and hollow squares represent the fast and slow elongating species, respectively), or to the superparamagnetic bead surface (blue), or when biotin-mDia1ΔN3 was directly attached to streptavidin-coated superparamagnetic bead surface (purple) or to an anti-DIG coated superparamagnetic bead surface through a torsionally unconstrained DNA linker (brown). Vertical error bars represent standard errors of mean (s.e.m) obtained from multiple data points obtained at the force. Horizontal error bars represent the 20% uncertainty in force calibration. The value labeled on each data point indicates the number of independent experiments carried out at the corresponding force. Inset shows the zoom-in of the slow elongating species. All of the data were collected in the presence of 500 nM G-actin. The solid black line is the predicted polymerization rate based on Eq. (1) with best fitting parameters Δ ≈ 5.36 nm, $\epsilon$ ≈ 1.21 *k*_B_ *T* and $k_{\mathrm{a}}^ \bullet (0) \approx 0.20$ nM^−1^ s^−1^ when $c_*^{ \bullet {\mathrm{0}}}$ is set as 400 nM. Several sample movies of superparamagnetic beads on elongating actin filaments are provided in Supplementary Movies 1–3. The elongation trajectory of an actin filament at two different forces corresponding to Supplementary Movies 1–3 are provided in Supplementary Figs. 5–7

In the case of GST-mDia1ΔN3-mediated actin polymerization, we found a fast elongating species (Figs. 2–4, red and blue solid squares in Fig. 4) and a slow elongating species (Fig. 4, red and blue hollow squares), both occupying a significant fraction. The data obtained from filaments with the barbed end tethered to glass surface through anti-GST (Fig. 4, red squares) are indistinguishable from the data obtained from actin filaments tethered to surface-immobilized NEM-HMM beads (Fig. 4, blue squares), indicating that the observed force-dependent behavior of the GST-mDia1ΔN3-mediated actin polymerization is independent on the approach used to immobilize the actin filament.

While the reason behind the existence of these two distinct elongating species is unclear, we believe that it might be related to the two different tethering cases (single-FH1 tethering or two-FH1 tethering) illustrated in Fig. 1a. The slower elongating species (Fig. 4, red and blue hollow squares) does not exhibit significant force dependence over 0–5 pN. We are particularly interested in the fast elongating species, because the elongating speed of this species is close to the speed of the protrusion of the leading edge of cells during rigidity sensing^23–25^ and this species exhibited strong force sensitivity as detailed below.

Figure 2a shows the movement of a bead attached to a fast elongating filament in the presence of 500 nM actin monomers along the direction of force at several different force levels, using GST-mDia1ΔN3 attached to the coverslip surface. The position of the bead at the beginning of the measurement was set as the origin. The data show that, at each force, the bead moved with an almost constant speed. Setting the starting of the data recorded at each force as time zero, the trajectories of the bead movement recorded at different forces can be plotted together and their speeds (i.e., the slopes) can be compared (inset). Data recorded from this example demonstrate that the actin polymerization speed increased by nearly 7-fold when force was increased from 1.3 ± 0.3 pN to 7.2 ± 1.4 pN (calibrated force at a given magnet-bead distance ±20% of relative uncertainty). In this example, force was increased from lower to higher levels. In Supplementary Fig. 3 we show that such force-dependent acceleration of actin elongation speed is independent from the force switching order. Figure 2b shows another example under the same reaction conditions. Increasing force from 3.2 ± 0.6 pN to 6.0 ± 1.2 pN resulted in a two-fold of increase in the polymerization speed. The bead movement stopped after washing away the free actin monomers, confirming that the bead movement was due to actin polymerization. Together, the results in Fig. 2 indicate the recruitment of actin monomers to the barbed end of actin filament through GST-mDia1ΔN3 is highly mechanosensitive.

Multiple independent experiments repeated at the same force under the same condition revealed a large variation in polymerization speed among different tethers. Figure 3 shows results from 17 independent experiments using different tethers, under a constant 1.9 ± 0.4 pN force in the presence of 500 nM actin monomers. The elongation speeds spread over a range from 20–127 nm s^−1^(7–47 subunits s^−1^), although two tethers produced an exceptionally fast speed that is several folds faster than the others.

### Force accelerates GST-mDia1ΔN3-mediated F-actin elongation

To see how reproducible the result in Fig. 2a could be, we measured the actin polymerization speeds for multiple tethers at several forces up to 9.2 ± 1.4 pN. At forces greater than 9 pN, the tethers broke in several seconds, preventing us from collecting enough data. Figure 4 shows the averaged speeds of GST-mDia1ΔN3-mediated actin polymerization in 500 nM G-actin at different forces, both when the GST-mDia1ΔN3 was attached to the coverslip surface (red) or to the superparamagnetic bead (blue). Increasing force from 1.3 ± 0.3 pN to 9.2 ± 1.4 pN accelerated actin polymerization of the force-sensitive species by several fold, and this speed reached a plateau of ~92 subunits s^−1^ (250 nm s^−1^) at forces above 6 pN.

### Biotin-mDia1ΔN3-mediated actin polymerization under force

We speculated that the emergence of the fast and slow elongating species observed in the GST-mDia1ΔN3-mediated F-actin polymerization experiments was caused by two different tethering possibilities (single-FH1 tethering or two-FH1 tethering as illustrated in Fig. 1a). We further hypothesized that the slow elongating species corresponds to two-FH1 tethering since this imposes a restriction on the relative rotation between the actin filament and the encircled FH2 ring at the barbed end. To test this possibility, we repeated the experiment using a biotin labeled mDia1ΔN3 to mediate F-actin polymerization (Fig. 1a, bottom left), where biotin-mDia1ΔN3 encircled on the barbed end of the filament was directly attached to a streptavidin-coated superparamagnetic bead. As a streptavidin has four biotin-binding states which can interact with the small biotin molecule located at the very end of the FH1 domain, we expected that two-FH1 tethering would be predominant. Consistent with this hypothesis, all the elongating filaments we observed in this experiments had speeds (Fig. 4, purple hollow squares) similar to the slow elongating species observed in the GST-mDia1ΔN3-mediated F-actin polymerization experiments.

In order to further confirm that the slow elongation was caused by the rotational constraint, we introduced a short dsDNA linker (445 bp)^26^ in between the biotin-mDia1ΔN3 and the superparamagnetic bead. The 5′ of one strand of the dsDNA linker was tagged with a biotin attached to the biotin-mDia1ΔN3 on the barbed end of the filament through a streptavidin, and the 5′ end of the complimentary strand was tagged with a digoxygenin attached to an anti-digoxygenin coated superparamagnetic bead. With this torsion-free dsDNA linker, the rotation between the FH2 ring of the biotin-mDia1ΔN3 and the actin filament becomes unconstrained regardless of single-FH1 or double-FH1 tethering. With the DNA linker attached, all the tethers observed in our experiments had elongation speeds similar to that of the fast species in the GST-mDia1ΔN3-mediated F-actin polymerization experiments, and this elongation could also be accelerated by increasing force (Fig. 4, brown solid squares). Together, these data suggest that the constraint on the relative rotation between the FH2 ring and the actin filament can significantly suppress actin polymerization.

### Mechanism of mDia1-mediated actin polymerization under force

**Fig. 5:**
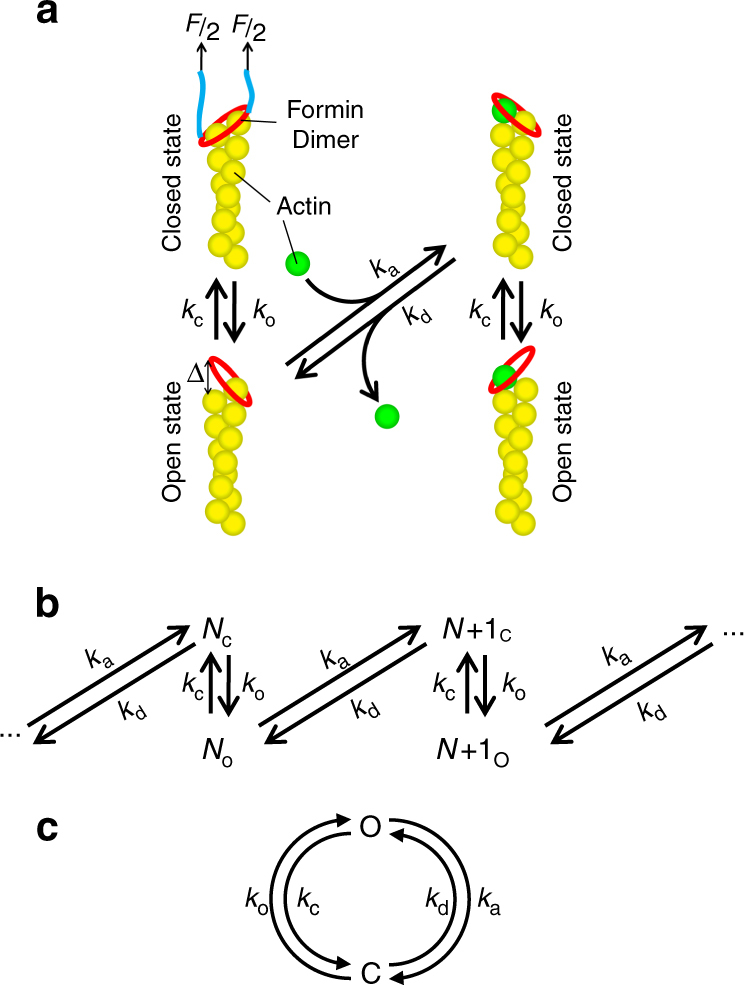
Kinetic model of FH2 dependent actin polymerization. **a** Schematics of the closed-open transition of FH2 associated at the barbed end and recruitment of actin monomers at the open state of the FH2 ring. **b** The corresponding transition diagram of actin elongation. **c** The corresponding transition diagram of recruiting one actin monomer, from which the polymerization speed can be derived

Our results suggest that the mDia1ΔN3-mediated actin polymerization is strongly sensitive to applied force in the scale of a few pN, when under rotationally unconstrained conditions. In this section we analyze a physical model that may explain this observation (Fig. 5). For simplicity, the model will be based on biotin-mDia1ΔN3-mediated actin polymerization with a torsionally unconstrained DNA linker, where both FH1 domains are linked to a streptavidin and are subject to force. We consider a simple two-state model where the FH2 ring can either be in the closed state or in the open state, and assume that actin monomers can associate with or dissociate from the barbed end of an actin filament only when the FH2 domain is in the permissive opened state denoted by • (Fig. 5a–c). A force-dependent net actin polymerization speed along the force direction, **v** = *v* **z**, can be written as:

1 $$v(F,c) = \frac{{\delta \tilde k _{\mathrm{a}}^ \bullet (0){\rm e}^{\beta F\delta }}}{{\left( {{\rm e}^{\beta \varepsilon } + {\rm e}^{\beta F\Delta /2}} \right)}}\left( {c - c_*^{ \bullet {\mathrm{0}}}{\rm e}^{ - \beta F\delta }} \right),$$

where *β* = (*k*_B_ *T*)^−1^, *k*_B_ is the Boltzmann constant and *T* is the temperature. $\epsilon$ denotes the energy cost of the transition from the closed state to the open state of the FH2 ring. *δ* ~ 2.7 nm is the filament extension increase by adding a new actin monomer to the barbed end, which is half of the size of one actin monomer (~5.4 nm)^15^. $c_*^{ \bullet {\mathrm{0}}}$ is the critical concentration of mDia1 associated barbed end of actin filament at zero force when the FH2 ring is in the open conformation.

*v*(*F*, *c*)/*δ* is the number of subunits recruited to the barbed end of the filament.

Equation (1) predicts that force speeds up *v*(*F*, *c*) until saturation if *δ* = Δ, or force speeds up *v*(*F*, *c*) over some force range then suppresses the polymerization at larger forces if *δ* < Δ. In addition, it predicts a force-dependent concentration of $c_*^ \bullet (F) = c_*^{ \bullet {\mathrm{0}}}{\rm e}^{ - \beta F\delta }$, consistent with that predicted in ref. ^18^. Here we emphasize that this is a general result from actin elongation under force, which is independent on assumptions made for the FH2 ring. The details of the derivation of Eq. (1) and the assumptions can be found in the Supplementary Note 1.

The equation contains four model parameters: $c_*^{ \bullet {\mathrm{0}}}$, Δ, $\epsilon$, and $k_{\mathrm{a}}^ \bullet (0)$. The critical concentration has been reported to be ~100 nM for the barbed end of naked actin filament^27^ and ~400 nM for mDia1 associated actin filament^28^. Therefore, $c_*^{ \bullet {\mathrm{0}}}$ should be somewhere between the two values. When $c_*^{ \bullet {\mathrm{0}}}$ is set as 400 nM, *v*(*F*, *c*)/*δ* is able to reasonably fit the averaged speeds of fast elongating filaments obtained from both GST-mDia1ΔN3 and biotin-mDia1ΔN3 with DNA linker at different forces (black solid line in Fig. 4), with the best fitting values for the remaining three parameters: Δ ≈ 5.36 nm, $\epsilon$ ≈ 1.21 *k*_B_ *T* and $k_{\mathrm{a}}^ \bullet (0) \approx 0.20$ nM^−1^ s^−1^. When $c_*^{ \bullet {\mathrm{0}}}$ is set as 100 nM, the best fitting parameters become Δ ≈ 5.49 nm, $\epsilon$ ≈ 1.8 *k*_B_ *T* and $k_{\mathrm{a}}^ \bullet (0) \approx 0.23$ nM^−1^ s^−1^. These results show that the predicted actin elongation rate has a very weak dependence on the value of $c_*^{ \bullet {\mathrm{0}}}$ in the range of 100–400 nM.

## Discussion

In summary, we show that mDia1ΔN3-mediated actin polymerization speed can be drastically accelerated by forces of a few pN, but only when the relative rotation between the FH2 ring and the encircled filament is not restricted. When such rotation is restricted, polymerization still occurred, but with an average speed at a few pN nearly 10-fold slower than when rotation was unconstrained, and polymerization rate also lost the sensitivity to the applied force. Similar force-dependent actin polymerization activity was also observed for Bni1(FH1-FH2)p (a.a.1227–1766, Supplementary Fig. 4), suggesting that this force dependence is likely conserved among different formins.

The actin elongation speed for the rotationally unconstrained filament could reach 10^2^ subunits per second at forces of a few pN in 500 nM G-actin monomers. This result implies that the G-actin on-rate was in the order of 10^2^ μM^−1^ s^−1^, which is >10 folds faster than the value (~11 μM^−1^ s^−1^) measured for association of G-actin to the free barbed end of actin filament in the absence of force and profilin^29^. On-rate faster than ~11 μM^−1^ s^−1^ was also reported in other single-molecule experiments. The experiment by Jégou et al.^15^ reported a net on-rate of 80 μM^−1^ s^−1^ at a force of around 3 pN for mDia1-mediated actin polymerization in the presence of profilin. Another experiment reported that CLIP-170 (a microtubule-binding protein) could bind tightly to mDia1, and the CLIP-170–mDia1 complex accelerates actin polymerization with a rate 18 times faster than free barbed end (~200 μM^−1^ s^−1^) in the absence of force^30^. Together, these experiments demonstrate that the on-rate of G-actin to the barbed end of actin filaments can be accelerated by multiple external factors such as tension, profilin, or other actin-binding proteins.

The force-dependent acceleration of the rotationally unconstrained filament was observed in the absence of profilin, which indicates that the FH2 homodimer encircled at the barbed end of the actin filament must be responsible for the force dependence of actin polymerization. Our theoretical analysis shows that the experimental results can be explained based on a force-dependent competition between association and dissociation of actin monomers at the barbed end of the actin filament that is encircled by a ring-like FH2 homodimer. Force biases the competition toward association by two energy saving factors, namely (1) reducing the critical concentration of net polymerization compared to the case without applied force, and (2) releasing the inhibition from the closed state of the FH2 ring.

The first factor arised as a natural result from the fact that recruitment of new actin monomers must lead to elongation along the force direction. The second factor results from the mechanical gating hypothesis of the FH2 ring, and in this case force contributes to a bias towards the open state of the ring. Our derivation is based on a reasonable assumption that there is a deformation between the closed and the open state, resulting in a positive extension change Δ along the force direction during a closed-to-open transition of the FH2 ring. Acceleration of actin polymerization based on mechanical deformation of the FH2 ring was first proposed by Kozlov et al ^18^. The derivation of the force-dependent actin polymerization speed in this study compliments that work by providing an analytical solution that can be directly compared with the single-molecule manipulation experiments.

Prior to this work, Courtemanche et al.^20^ investigated the effects of force on budding yeast formin Bni1p-dependent actin polymerization. In the absence of profilin, the authors found that force suppressed actin polymerization at sub pN forces, which contradicts to the observations in our study obtained based on using formin mDiaΔN3 and Bni1(FH1-FH2)p (a.a.1227–1766). In their experiments, biotin-tagged Bni1p molecules were anchored to streptavidin molecules on a lipid bilayer embedded with rigid diffusion barriers. Force was applied by flow-stretching of the filament. In flow, the filament is rotated around the anchoring point toward the surface, which might press the filament against the barrier, causing a rotational constraint that might explain this discrepancy.

A previous study by Jégou et al.^15^ using mDia1 shows much less extent of the force-dependent acceleration of actin elongation than in this study. However, that experiment was carried out in the presence of profilin, which is different from our experiments done in the absence of profilin. Therefore, the difference can be explained by the presence/absence of profilin in solution. A more recent study by Kubota et al.^31^ reported force-dependent acceleration of mDia1ΔN3-mediated actin polymerization for both ATP-G-actin and ADP-G-actin, in the presence or absence of profilin. Under the condition of ATP-G-actin in the absence of profilin, they reported a very weak force dependence of actin polymerization (~11 subunits per second at ~4 pN) similar to the slower species observed in our experiments (Fig. 4, hollow squares). In that work, the experiments were carried out using dual-trap optical tweezers, where the bead-to-bead distance was increased manually to maintain a roughly constant force. The rate of bead-to-bead distance increase was interpreted as the actin elongation speed at the corresponding forces. Such measurement would depend on how fast the bead-to-bead distance was changed. Based on the Supplementary Fig. 2 in ref. ^31^, the typical rate of change of the bead-to-bead distance was in the range of 20–30 nm s^−1^. At this speed, only the slower species could build forces of a few pN that were detected in experiments. In contrast, forces built in the faster species would be less than 1 pN (Fig. 4, solid squares), which might be the reason why it was not detected in that work.

For the fast elongating filaments obtained under rotationally unconstrained conditions, we observed a large, several-fold variation in the polymerization speeds among different tethers under the same actin monomer concentration and force. We reason that the intrinsic uncertainty of force calibration by approximately ~20% may be one of the causes of this variation of polymerization speed (Supplementary Note 3). In fact, the highly sensitive force dependence (Fig. 4, black solid curve) predicted by Eq. (1) results in significant variation of force-dependent polymerization speeds when a ~20% force uncertainty is considered. However, this factor alone is insufficient to explain the level of variation in the polymerization speeds observed in experiments (Supplementary Fig. 1). Another possibility is that the streptavidin-coated superparamagnetic bead might slide on the actin filament, or in the case of actin filament trapped by NEM-HMM-coated beads, the filament might slide over the NEM-HMM coated beads. This possibility requires stochastic dissociation of actin filament from a streptavidin or NEM-HMM-coated bead, which must be re-caught by the same (or another) streptavdin or NEM-HMM molecule on the same bead. Although we cannot completely exclude this possibility, such events must be rare since once detached, the filament would drift away at a speed >10 μm s^−1^ in our force range (Supplementary Fig. 2). In addition, should such slippage events happen, one would expect large stepwise jumps in extension, which were not observed in our experiments. Therefore, this large variation in polymerization speeds is likely due to other factors, which warrants future studies.

The capability of the FH2 homodimer of mDia1 to sense force enables it to accelerate actin polymerization by a few folds upon a few pN force increase, as suggested by this work. Forces of a few pN are in a range that can be produced by a single-myosin motor^32^ and by microtubule protrusion^33,34^. Besides force generated by motor molecules, large force-bearing proteins that contain a tandem repeats of subdomains, which are often present as components in the force transmission pathway in cytoskeleton network, can also buffer force over a similar range due to stochastic unfolding and refolding of the domains under force^35^. Therefore, the force range in which the mDia1-mediated actin polymerization shows mechanosensitivity is highly physiologically relevant. Accelerated actin polymerization in response to actomyosin contraction can act as a mechanical absorber, preventing fast accumulation of tension that may disrupt the filament.

Our results show that the force-dependent mDia1-mediated actin polymerization requires unconstrained relative rotation between the FH2 ring and the actin filament it encircles. It is likely that in living cells mDia1 is linked to other cellular structures through flexible adapter proteins that can rotate freely (as proposed in ref. ^21^). Importantly, formin can bind to cytoplasm membrane indirectly through other adapter proteins. For example, mammalian formin-1 and formin-like 2 can bind *α*-catenin^36,37^, while *α*-catenin is known to form a heterodimer with *β*-catenin and together they bind the intracellular tail of the E-cadherin at cell–cell adherence junction^38^. Due to the fluidity of lipid membrane, membrane anchored formin should be able to rotate; therefore the actin filament polymerized from such membrane anchored formin is likely torsionally unconstrained.

In our experiment, the method of anchoring mDia1 to the surface also results in the FH1 domains being under force. The FH1 domain provides binding sites for profilin through its polyproline tracts, and this interaction is also likely to be mechanically regulated. Since our study focuses solely on the understanding of the mechanosensitivity of the FH2 homodimer, profilin was not introduced in order to avoid complications from the possible mechanosensitive interaction between FH1 domains and profilin/actin complex. To fully understand the mechanosensitive regulation of actin polymerization by mDia1, the effects of force on the activities of both FH2 and FH1 domains and the interplay between the two domains under force must be studied. Therefore, our ongoing studies will aim to quantify the affinity between FH1 and profilin/actin complex and the transfer of profilin/actin complexes from the FH1 domains to the barbed end of actin filaments encircled by FH2 homodimers when the FH1 domains are subject to force.

## Methods

### Materials

Actin was purified from rabbit muscle, stored in G-Ca buffer(2 mM Tris, pH 8.0, 0.5 mM DTT, 0.2 mM ATP, 0.1 mM CaCl_2_, 0.01% sodium azide, 10% glycerol) at −80 °C. Formin mDia1ΔN3(GST-avi-FH1-FH2) was expressed in Rosetta cells and purified, stored in buffer(50 mM Tris pH 7.0, 50 mM KCl, 1 mM EGTA, 1 mM DTT, 0.01% sodium azide, 10% glycerol) at −80 °C. Formin Bni1(FH1-FH2)p (GST-FH1-FH2, a.a. 1227–1766) was expressed in Rosette 2 cells and purified, stored in buffer (25 mM Tris pH 7.4, 0.5 mM EDTA, 500 mM NaCl, 4 mM DTT) at −80 °C. Biotinylated-mDia1ΔN3 was obtained by BirA biotin ligase reaction, and the GST tag was digested by thrombin before BirA biotin ligation. HMM was kindly provided from the lab of Dr. James Sellers, NIH. HMM was treated with NEM and stopped with DTT.

### Sample preparation

Four different tethering methods were used in our experiments (Fig. 1a), which required different preparations of coverslip, seed actin filaments, and superparamagnetic beads. These preparations are described in detail in Supplementary Methods.

### Experimental procedure

In order to perform the measurements, the seeding actin filaments were incubated in the channel for 10 min to allow actin filaments seeds to bind to the surface. After removal of free actin filaments by washing the channel with KMEI buffer (10 mM imidazole, 50 mM KCl, 1 mM MgCl_2_, 1 mM EGTA, 400 μM ATP, 0.5 mM DTT), superparamagnetic beads were introduced into the channel, and incubated for 5 min to allow them to bind to actin filaments. Finally, 500 nM G-actin was flowed into the channel in KMEI buffer for actin elongation. Force was applied to the tether through the attached superparamagnetic beads. The elongation of the filament was indicated by the movement of the tethered superparamagnetic bead in real time.

### Magnetic tweezers

The transverse magnetic tweezers using in this study was made in house, and controlled as described in ref. ^39^. This set-up can apply force to a superparamagnetic bead attached to the filament by a pair of permanent magnets placed outside the reaction chamber. Force can be generated with a small angle above the focal plane (Supplementary Note 2). The stage was mounted on a step motor controlled with a MP285 manipulator (Shutter), which can move in 3-dimensions with stepping accuracy of 40 nm. When the superparamagnetic bead attached to a filament moved off the viewing area, the stage could be moved back so the elongation of the filament could be continuously tracked. The superparamagnetic beads were imaged with 50X magnification at a sampling rate of 100 Hz, with its position determined by the centroid.

### Force calibration

For a given superparamagnetic bead, the force applied to the bead *F*(*s*), depends on the magnet-bead separation *s*. At a given magnet-bead separation, different superparamagnetic beads may feel a different force, *F*_*i*_(*s*), due to different levels of magnitude of magnetization. The force ratio of two beads, *F*_2_(*s*)/*F*_1_(*s*), equals the ratio of the maximal magnetization of the two beads, $M_1^0{\mathrm{/}}M_2^0$. For the M270 superparamagnetic Dynal beads (Invitrogen) used in this study, we found that the histogram of the pairwise force ratio built from 100 beads, $\alpha _{ij} = F_i(s){\mathrm{/}}F_j(s) = M_i^0{\mathrm{/}}M_j^0$, when peaked at 1 has a standard deviation of *σ* = 0.2. These calibration results suggest that for any given bead, the force can be estimated as: $F_i(s) = (1 + \sigma )\bar F(s)$, where $\bar F(s)$ is the average force over many (>20) beads at the same magnet-bead separation *s*. In other words, for any bead we can estimate the force by $\bar F(s)$ with a relative uncertainty of ~20%. $\bar F(s)$ was obtained by recording the drift speed of beads in 90% (volume fraction) glycerol water solution at 25°, with an expected viscosity of *η* = 0.21 Pa·s^40^. During the measurements, the beads were at least 5 μm above the surface to avoid hydrodynamic coupling between the bead and the surface. Therefore its drag coefficient can be calculated with the Stokes law *ξ* = 6*πηr*, where *r* = 1.4 or 0.5 μm is the radius of the beads. Drift speeds *v*_*i*_(*s*) were recorded for more than 20 beads indexed by *i* at various values of magnet-bead separation, and forces were obtained by *F*_*i*_(*s*) = *ξv*_*i*_(*s*).

### Solution exchange

A 100 μm thick polymer (OF-134-V2, Mypolymers) membrane with an array of rectangular through holes (0.2 mm × 2 mm) was used for eliminating flow perturbation during solution exchange, which was slightly revised from the 50 μm-diameter circular hole array described in our previous publication^41^, in order to increase the chance to find tethers in the holes.

### Data availability

The authors declare that all data supporting the findings of this study are available within the article and its Supplementary Information or from the corresponding author upon reasonable request.

## Electronic supplementary material

Supplementary Information

Description of Additional Supplementary Files

Supplementary Movie 1

Supplementary Movie 2

Supplementary Movie 3
