# Supplementary material for: mDia1 senses both force and torque during F-actin filament polymerization
Source: Nat Commun. 2017 Nov 21;8:1650. doi: 10.1038/s41467-017-01745-4 (PMC5698482; doi:10.1038/s41467-017-01745-4)
Supplement: Supplementary file 1 — Supplementary Information [file 41467_2017_1745_MOESM1_ESM.pdf]

I. Supplementary Figures

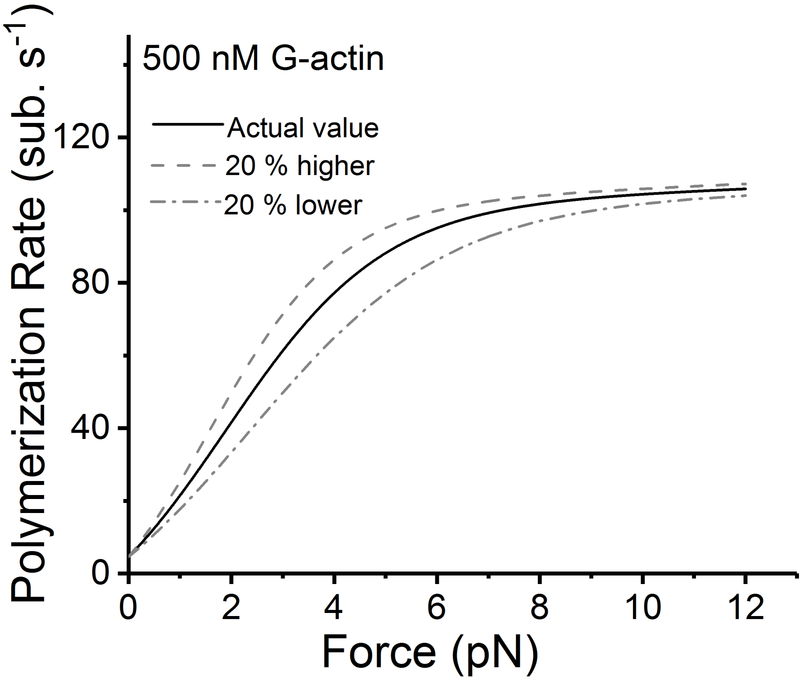

Supplementary figure 1. The predicted extent of contribution from the 20% uncertainty in force calibration to the variation of force-dependent polymerization speeds.

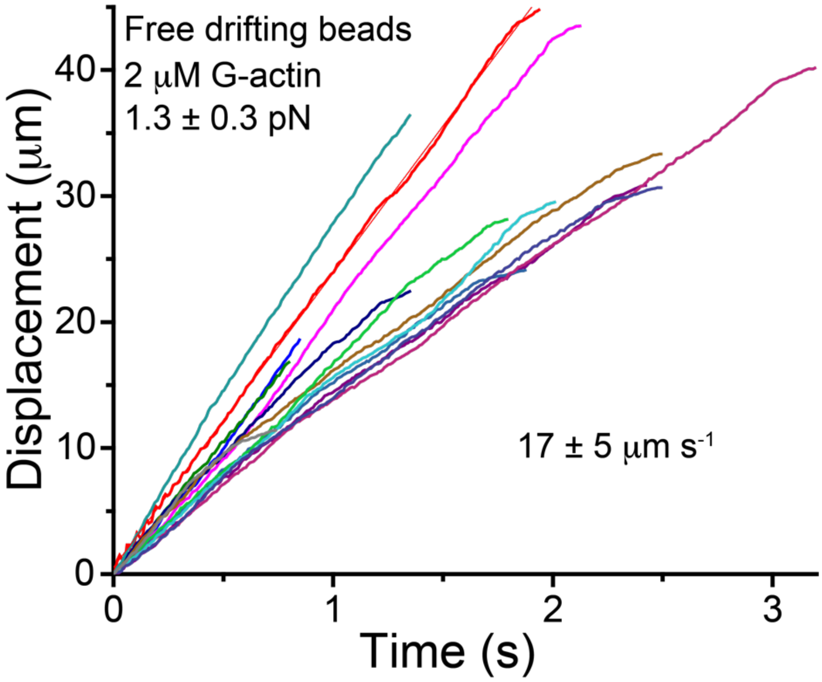

Supplementary figure 2. Displacement of free drifting beads. Data show the displacement of multiple free drifting beads near coverslip surface at forces of 1.3±0.3 pN. From the slope of the displacement of the beads as a function of time, the average speeds of these beads were determined as ~17 $\mu m\ s^{-1}$, which is about 100 times faster than the movement speed of beads attached to elongating actin filaments. 19 free beads were tested.

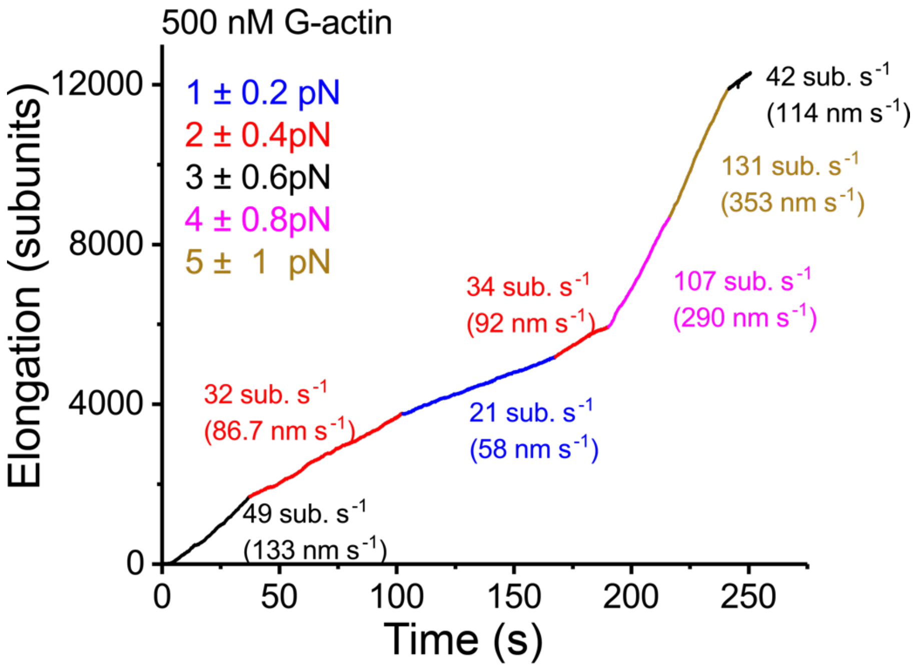

Supplementary figure 3. mDia1ΔN3-mediated actin polymerization rate of a single filament at several forces. Data show that the force-dependent polymerization rate is insensitive to the force changing order.

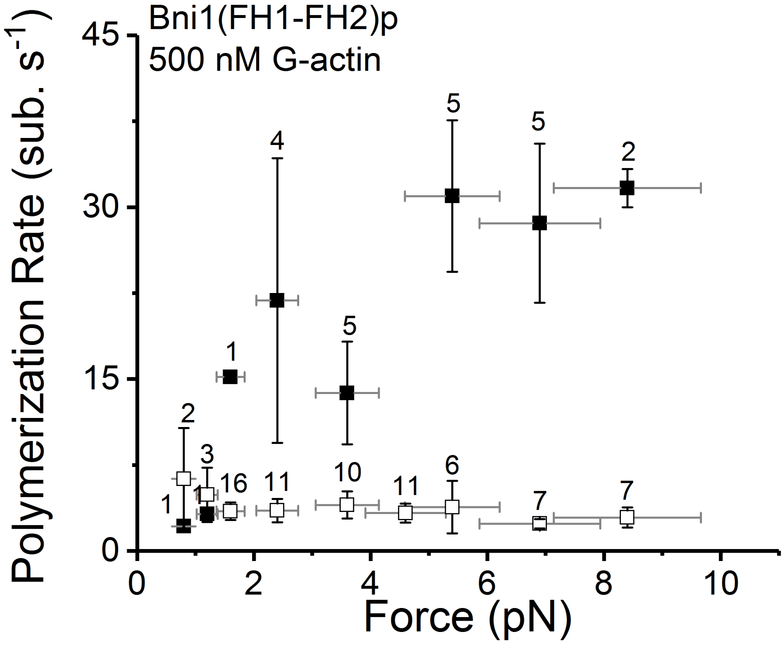

Supplementary figure 4. Force-dependent polymerization rate of Bni1(FH1-FH2)p mediated actin polymerization. Data show that the force-dependence of Bni1(FH1-FH2)p mediated actin polymerization also has a force-sensitive species (solid squares) and a force-insensitive species (hollow squares), similar to that observed for mDia1-mediated polymerization. Vertical error bars represent standard errors of mean obtained from multiple data points obtained at the force. Horizontal error bars represent the 20% uncertainty in force calibration. The value labelled on each data point indicates the number of independent experiments carried out at the corresponding force. All of the data were collected in the presence of 500 nM G-actin.

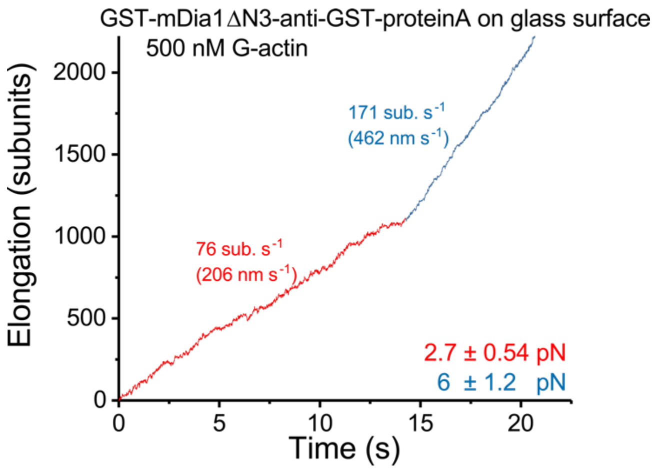

Supplementary figure 5. The elongation trajectory of an actin filament at two forces (~ 2.7 pN (red) and ~ 6 pN (blue))corresponding to Supplementary Movie 1.

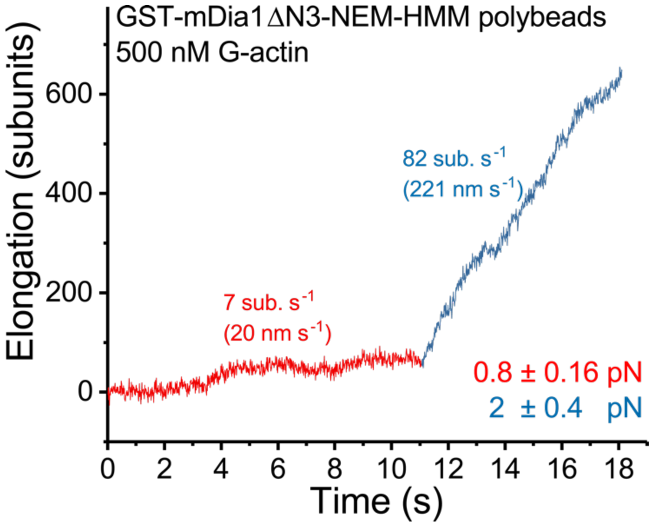

Supplementary figure 6. The elongation trajectory of an actin filament at two forces (~ 0.8 pN (red) and ~ 2 pN (blue))corresponding to Supplementary Movie 2.

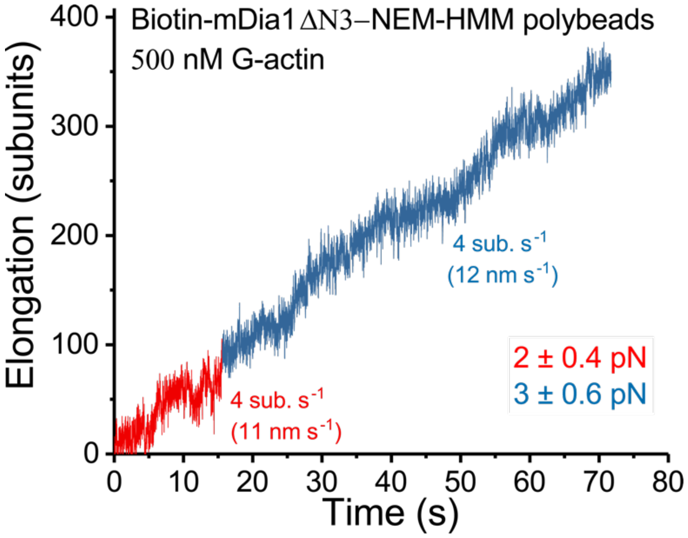

Supplementary figure 7. The elongation trajectory of an actin filament at two forces (~ 2 pN (red) and ~3 pN (blue))corresponding to Supplementary Movie 3.

Supplementary Note 1

Actin polymerization model - We assume that the rate of association and dissociation is scaled by the probability of the FH2 in the open state. We further assume that the open state involves a partially detached FH2 ring, which may undergo force-dependent conformational fluctuations. The elasticity of the open state is described by a force-dependent extension $\Delta(F)$ (Fig. 5 in the main text). Under a constant applied force F, due to the parallel linkages of the FH1 domains, the partially detached part of the FH2 ring is approximately subject to a force of $F/2$ (Fig. 5 in the main text), which contributes to a force-dependent conformational entropic free energy of $\phi_{\mathrm{FH2}}(F/2) = -\int_0^{F/2} \Delta(f')df'$. This energy equals $-F\Delta/2$ when $\Delta(f')$ is a constant. The force-dependent probability of the $\bullet$ state becomes:

$$p^{\bullet}(F) = e^{-\beta\phi_{\mathrm{FH2}}(F/2)} / \left(e^{\beta\epsilon} + e^{-\beta\phi_{\mathrm{FH2}}(F/2)}\right),$$

where $\beta = (k_{\mathrm{B}}T)^{-1}$, k_{B} is the Boltzmann constant and T is the temperature. ϵ denotes the energy cost of the close-to-open transition of the FH2 ring.

By denoting $k_{\mathrm{a}}^{\bullet}(F)$ and $k_{\mathrm{d}}^{\bullet}(F)$ as the force dependent association and dissociation rates from the barbed end when FH2 is in the $\bullet$ state, the force-dependent association flux and dissociation flux are $r_{\mathrm{a}}(F,c) = p^{\bullet}(F)k_{\mathrm{a}}^{\bullet}(F,c)$ and $r_{\mathrm{d}}(F,c) = p^{\bullet}(F)k_{\mathrm{d}}^{\bullet}(F,c)$. Their ratio is:

$$\frac{r_{\mathrm{a}}(F,c)}{r_{\mathrm{d}}(F,c)} = \frac{k_{\mathrm{a}}^{\bullet}(F,c)}{k_{\mathrm{d}}^{\bullet}(F,c)} = \frac{k_{\mathrm{a}}^{\bullet}(0,c)}{k_{\mathrm{d}}^{\bullet}(0,c)} e^{\beta F\delta},$$

where $\delta \sim 2.7$ nm is the filament extension increase by adding a new actin monomer to the barbed end, which is half of the size of one actin monomer (5.4 nm). The factor of $e^{\beta F\delta}$ is the result of

the net extension increase due to recruitment of a new actin monomer that reduces the potential energy by an amount of $-F\delta$ after one reaction cycle in the kinetics diagram shown in Fig. 5.

$k_{\text{a}}^{\bullet}(F,c)$ is the association rate to the barbed end with a constitutively open FH2 ring. Force changes the energy barrier of adding an actin monomer by an amount of $-\int_{0}^{F/2}(2\delta-\Delta(f'))df'$. Applying Arrhenius' law,

$$k_{\text{a}}^{\bullet}(F,c)=k_{\text{a}}(0,c)e^{\beta(F\delta+\phi_{\text{FH2}}(F/2))}.$$

The polymerization speed is therefore:

$$\begin{aligned} v(F,c) &= \delta\left(r_{\text{a}}(F,c)-r_{\text{d}}(F,c)\right) \\ &= \delta r_{\text{a}}(F,c)\left(1-\frac{k_{\text{d}}(0,c)}{k_{\text{a}}(0,c)}e^{-\beta F\delta}\right) \\ &= \frac{\delta k_{\text{a}}^{\bullet}(F,c)e^{-\beta\phi_{\text{FH2}}(F/2)}}{\left(e^{\beta\epsilon}+e^{-\beta\phi_{\text{FH2}}(F/2)}\right)}\left(1-\frac{k_{\text{d}}^{\bullet}(0,c)}{k_{\text{a}}^{\bullet}(0,c)}e^{-\beta F\delta}\right) \\ &= \frac{\delta\tilde{\kappa}_{\text{a}}^{\bullet}(0)e^{\beta F\delta}}{\left(e^{\beta\epsilon}+e^{-\beta\phi_{\text{FH2}}(F/2)}\right)}\left(c-c_{*}^{\bullet 0}e^{-\beta F\delta}\right). \end{aligned}$$

In the last line, we have assumed that the dissociation rate $k_{\text{d}}^{\bullet}(0,c)=k_{\text{d}}^{\bullet}(0)$ is concentration independent and the association rate is proportional to concentration, $k_{\text{a}}^{\bullet}(c)=\tilde{\kappa}_{\text{a}}^{\bullet}(0)c$. $c_{*}^{\bullet 0}=k_{\text{d}}^{\bullet}(0)/\tilde{\kappa}_{\text{a}}^{\bullet}(0)$ is the critical concentration of mDia1 associated barbed end of actin filament at zero force when the FH2 ring is in the open conformation.

As revealed in the above equation, $v(F,c)$ depends on the elasticity of the FH2 ring described by $\phi_{\text{FH2}}(F)$. Eq. (1) in the main text is recovered in the simplest case where $\Delta(f)$ is a constant.

Supplementary Note 2

Stretching angle calibration - The force range used in studies of actin polymerization is from 0.5 pN to 10 pN. At the corresponding magnet-bead separation range, we tune the height of the magnet relative to the coverslip such that the height of a free bead increases 5-8 μm after drifting along the force direction over a distance of 50 μm, which corresponds to a stretching angle range of roughly $6 - 10$ degrees.

Supplementary Note 3

Contribution of the 20% *uncertainty in force calibration to the variation of force-dependent polymerization speeds* - Eq. (1) predicts a highly sensitive force dependence of the polymerization speed of mDia1ΔN3 mediated actin polymerization. Considering that there is 20% uncertainty in force calibration, there may be a large variation of polymerization speeds observed in experiments at the same force. Supplementary Figure 1 plots the level of the variations of the polymerization speeds that can be explained by the 20% uncertainty in force calibration. The solid line predicted by Eq. (1), and the dashed line and the dash-dotted line are the speeds assuming that the force is lower (dashed) or higher (dash-dotted) than actual value by 20%. The results show that, although the 20% uncertainty in force calibration indeed contributes a significant variation in force-dependent polymerization speeds, this factor alone is not able to explain the level of the variation observed in experiments.

Supplementary Methods

Preparation of coverslip, seed actin filaments, and superparamagnetic beads - Four different tethering methods were used in our experiments (Fig. 1A), which required different preparations of coverslip, seed actin filaments, and superparamagnetic beads as described below.

In experiments using a protein A-antiGST-GST-mDia1ΔN3-actin-superparamagnetic bead tether, the coverslip was functionalized with protein A. To functionalize the coverslip, the coverslip was sonicated with 50 % detergent (Decon 90) for 30 minutes and then with acetone for 20 minutes, before being dried in an oven and plasma cleaned for 10 minutes. The coverslip was then incubated with 1% APTES in methanol for 1 hour at room temperature, washed with distilled water and dried in an oven. Next, the coverslip was made to be part of a channel, then incubated with 0.3 % glutaraldehyde in 2× PBS for 6 hours at room temperature, then rinsed with 1×PBS. Following glutaraldehyde treatment, the coverslip was incubated overnight with 50 μg/mL protein A (Sigma P6031) in 1× PBS at room temperature, and then blocked overnight with 5 % BSA in 1×PBS at room temperature. To prepare the GST-mDia1ΔN3 capped actin filaments, GST-mDia1ΔN3 was first incubated with anti-GST antibody(Sigma-G7781) for 1 hour on ice, at a ratio of 1:1. The seeding actin filaments were polymerized by addition of 2 μM G-actin, 200 nM biotinylated actin, and 750 nM GST-mDia1ΔN3-anti-GST antibody complex in KMEI buffer (10 mM imidazole, 50 mM KCl, 1 mM $MgCl_2$, 1 mM EGTA,400 μM ATP, 0.5 mM DTT) for 20 minutes at room temperature, and stabilized with 2 μM Phalloidin for 10 minutes at room temperature. The superparamagnetic beads used for the experiments were streptavidin-coated M270 or MyOne beads (Invitrogen).

In experiments using a NEM-HMM-actin-GST-mDia1ΔN3-antiGST-proteinA-superpara- magnetic beads tether, NEM-HMM functionalized polystyrene beads on the coverslip surface were used. To prepare the beads, 3-μm-diameter polybeads(17134, Polyscience) were incubated overnight with NEM-HMM at 4 °C, and then blocked with 5 % BSA in 1$\times$PBS. NEM-HMM beads were non-specifically bound to the cleaned glass surface in the channel by simply incubating. The seeding actin filaments were polymerized with 750 nM GST-mDia1ΔN3, 2 μM G-actin in KMEI buffer, which were then stabilized with 2 μM Phalloidin. To prepare the antiGST-protein A coated superparamagnetic beads, streptavidin coated superparamagnetic beads were incubated with 50 μg/mL biotin-protein A (Sigma-P2165) in 1$\times$ PBS overnight at room temperature, then washed in 1$\times$ PBS, and incubated overnight with 50 μg/mL anti-GST antibody in 1$\times$ PBS at room temperature, followed by another wash before blocking in 5 % BSA in 1$\times$PBS. In experiments using a NEM-HMM-actin-GST-Bni1(FH1-FH2)p-antiGST-proteinA-superparamagnetic beads tether, the seeding actin filaments were polymerized with 750 nM GST- Bni1(FH1-FH2)p, 2 μM G-actin in KMEI buffer. Other preparation were same as above.

In experiments using a NEM-HMM-actin-biotin-mDia1ΔN3-superparamagnetic beads tether, NEM-HMM functionalized polystyrene beads and superparamagnetic beads as described above were used. The seeding actin filaments were polymerized by 750 nM biotin-mDia1ΔN3, 2 μM G-actin in KMEI buffer and stabilized with 2 μM Phalloidin.

In experiments using a NEM-HMM-actin-biotin-mDia1ΔN3-streptavidin-biotin-DNA-DIG-antiDIG-superparamagnetic beads tether, NEM-HMM functionalized polystyrene beads and the

seeding actin filaments polymerized with biotin-mDia1ΔN3 as described above were used. The seeding filaments were incubated with streptavidin so that biotin-mDia1ΔN3 were attached to streptavidin molecules. The anti-DIG coated superparamagnetic beads were prepared as follows: 1 μm-diameter carboxyl superparamagnetic beads (MyOne, Invitrogen) were treated with EDC and NHS in MES buffer (pH 6.0) for 30 mins at room temperature, then rinsed with 2$\times$ PBS, incubated overnight with 50 μg/mL anti-DIG in 1$\times$ PBS at room temperature, and blocked in 5 $\%$ BSA in 1$\times$ PBS. The anti-DIG superparamagnetic beads were then incubated with 445 bp DNA labelled with a DIG on one strand at one end and a biotin on the complimentary strand at the other end for 20 minutes at room temperature. Later, these beads were flowed into channels to search for the streptavidin attached biotin-mDia1ΔN3 on the actin filaments.
