# Supplementary material for: mDia1 senses both force and torque during F-actin filament polymerization
Source: Nat Commun. 2017 Nov 21;8:1650. doi: 10.1038/s41467-017-01745-4 (PMC5698482; doi:10.1038/s41467-017-01745-4)
Supplement: Supplementary file 2 — Description of Additional Supplementary Files [file 41467_2017_1745_MOESM2_ESM.pdf]

Description of Additional Supplementary Files

File Name: Supplementary Movie 1
Description: Supplementary Movie 1 was recorded for a filament tethered between glass surface (through protein A-anti-GST-GST-mDia1ΔN3) and streptavidin coated superparamagnetic beads. Three beads were attached to the filament, so the force is three times of that applied to a single bead at the same magnet-bead distance.

File Name: Supplementary Movie 2
Description: Supplementary Movie 2 was recorded for a filament tethered between a NEM-HMM polystyrene bead stuck on surface and an anti-GST coated superparamagnetic bead at the GST-mDia1ΔN3 associated barbed end of the filament.

File Name: Supplementary Movie 3
Description: Supplementary Movie 3 was recorded for a filament tethered between a NEM-HMM polystyrene bead stuck on surface and a streptavidin coated superparamagnetic bead at the biotin-mDia1ΔN3 associated barbed end of the filament.
